# Establishment of an enzyme-linked immunosorbent assay for mouse pancreatic polypeptide clarifies the regulatory mechanism of its secretion from pancreatic γ cells

**DOI:** 10.1371/journal.pone.0269958

**Published:** 2022-08-17

**Authors:** Daisuke Saito, Yuko Nakagawa, Takashi Sato, Ayako Fukunaka, Ofejiro Blessing Pereye, Nobuhiro Maruyama, Hirotaka Watada, Yoshio Fujitani

**Affiliations:** 1 Laboratory of Developmental Biology & Metabolism, Institute for Molecular and Cellular Regulation, Gunma University, Maebashi, Gunma, Japan; 2 Department of Metabolism & Endocrinology, Juntendo University Graduate School of Medicine, Tokyo, Japan; 3 Immuno-Biological Laboratories Co., Ltd., Fujioka, Gunma, Japan; 4 Center for Therapeutic Innovations in Diabetes, Juntendo University Graduate School of Medicine, Tokyo, Japan; 5 Center for Identification of Diabetic Therapeutic Targets, Juntendo University Graduate School of Medicine, Tokyo, Japan; 6 Sportology Center, Juntendo University Graduate School of Medicine, Tokyo, Japan; International University of Health and Welfare School of Medicine, JAPAN

## Abstract

Pancreatic polypeptide (PP), secreted from γ cells of the islets of Langerhans, is a 36 amino-acid peptide encoded by the *Ppy* gene. Although previous studies have reported that PP causes a decrease in appetite, the molecular mechanism that regulates PP secretion has not been fully elucidated. Lack of understanding of the regulatory mechanism of PP secretion may be partially owing to the lack of assay systems that can specifically detect PP. We recently developed the mouse monoclonal antibody 23-2D3 that specifically recognizes PP. In the present study, we developed a sandwich enzyme-linked immunosorbent assay for the measurement of mouse PP, and directly monitored intracellular Ca^2+^ concentrations in *Ppy*-expressing cells from a newly developed reporter mouse. Using these systems, we identified agonists, such as carbachol and glucose-dependent insulinotropic polypeptide (GIP), which stimulate PP secretion. We further demonstrated that, unlike the case of GIP-induced insulin secretion from β cells, there is a unique mechanism by which PP secretion is triggered by an increase in intracellular Ca^2+^ concentrations via voltage-dependent calcium channels even in low-glucose conditions.

## Introduction

The islets of Langerhans in the pancreas are composed of four major types of endocrine cells with different properties. Pancreatic polypeptide (PP) is a peptide consisting of 36 amino acid residues [1–3], and is secreted by specialized pancreatic islet cells, i.e., PP cells (also known as F cells or γ cells). γ cells are located at the periphery of pancreatic islets in mice, and mainly in islets in the head region of the pancreas [4]. As a pancreatic endocrine cell, γ cells have been less studied compared with β and α cells, but is known to affect gastrointestinal motility, glucose homeostasis, and appetite regulation [5].

PP belongs to the NPY family of peptides [5], and has high homology with the other family members, i.e., neuropeptide Y (NPY) and peptide YY (PYY). The NPY family commonly acts on seven transmembrane G protein-coupled Y receptor superfamily proteins (Y1, Y2, Y4, Y5 and y6 in mice) [5–8], and modulates food intake by affecting central neural circuits [8–10]. Whereas PP binds to all members of the Y receptor superfamily, it shows greatest affinity to Y4, and activates hypothalamic nuclei that are crucial in the regulation of appetite and satiety. Indeed, previous reports have shown that the administration of PP to mice suppresses feeding, reduces body weight, increases energy consumption, and improves insulin resistance and dyslipidemia caused by obesity [10–13]. Studies using knockout mice lacking the Y4 receptor demonstrated that the Y4 receptor is required for the inhibitory effect of PP on food intake [10]. However, the genetic deletion of *Ppy*, which encodes PP, does not alter the body weight of mice [14], suggesting that other peptides compensate for the role of *Ppy* in the body weight control of *Ppy*^-/-^ mice. Regarding human diseases, it is known that patients with Prader-Willi syndrome, a genetic disorder characterized by overeating and morbid obesity, have decreased basal serum PP levels. Importantly, it has been reported that when PP is administered intravenously to patients with Prader-Willi syndrome, serum PP levels return to normal, and food intake is substantially reduced [15].

Previous studies on the regulation of PP secretion have focused mainly on the effects of PP on feeding control, and the involvement of a number of regulators has been investigated, based on a system of measuring PP by radioimmunoassay (RIA) [16–20]. These studies suggested that PP secretion is postprandially increased [21], and is stimulated by a variety of signaling factors acting in response to food intake, including carbachol, arginine, lipids, G-protein coupled receptor 120 agonists, and the incretin hormone GIP [16–20,22]. However, many previous reports on the regulation of PP secretion have been controversial. In particular, some studies have reported that PP secretion is directly stimulated by glucose [23], whereas other studies have reported that PP secretion is not directly stimulated by glucose [16]. Furthermore, although most of　the circulating PP in blood is released from pancreatic γ cells, the regulatory and signaling mechanisms of PP secretion remain unclear.

One of the main reasons for the inconsistent results described above may be the lack of antibodies that specifically recognize PP and the absence of an enzyme-linked immunosorbent assay (ELISA) that can accurately measure PP concentrations. In particular, many PP antibodies that have been used in ELISAs cross-react with PYY, which is the NPY family protein that is most closely related to PP, making it difficult to clarify accurate changes in PP concentrations [24,25]. To solve this problem, we recently developed and reported a unique PP monoclonal antibody (23-2D3), and demonstrated its usefulness for specific detection of PP [24]. In the present study, we developed a new sandwich ELISA for PP using this 23-2D3 antibody, and reassessed the regulatory mechanism of PP secretion from γ cells.

## Materials and methods

### Animal care

The protocol for animal experiments in this study has been approved by the Animal Care and Ethical Committee at Gunma University (Permission Number: D21-040). Mice were euthanized by cervical dislocation after anesthesia with isoflurane (Fujifilm, Osaka, Japan), using an inhalation rodent anesthesia system (NARCOBIT-E, Natsume Seisakusho Co., Ltd., Tokyo, Japan) and all efforts were made to minimize suffering. All mice were housed in specific pathogen-free barrier facilities, maintained under a 12-hour light/dark cycle, and provided standard rodent food (Oriental Yeast, Tokyo, Japan) and water ad libitum.

### Animal model

*Ppy*^Cre/Cre^ (*Ppy*-KO) mice, in which the protein-coding region of *Ppy* in exon 2 is replaced by a nuclear translocation signal (NLS) -Cre, were established previously [24]. *Ppy-Clover-PEST* knock-in mice were generated using CRISPR-Cas9 system as described in S1A Fig [26–28].

### ELISA system for quantification of PP

First, rabbit polyclonal antibodies to the C-terminal region of human PP were prepared. Rabbits were immunized with a synthetic peptide (CDLRRYINMLTRPRY-NH_2_) which was coupled with keyhole limpet hemocyanin as a carrier protein. This peptide sequence corresponds to region D22 to Y36 in human PP. The immunoglobulin G (IgG) fractions against this peptide sequence was obtained from the sera of immunized rabbits using columns containing the antigen coupled with activated Thiol Sepharose 4B beads (GE Healthcare Bio-Sciences AB, Uppsala, Sweden). The resulting purified IgG, which was named PoAb-C, was then biotinylated using EZ-Link Sulfo-NHS-LC-Biotin (Thermo Fisher Scientific, Tokyo, Japan) according to the manufacturer’s protocol.

Subsequently, a sandwich PP ELISA, which is a combination of two antibodies (PoAb-C and 23-2D3) was developed. The specificity of the mouse monoclonal antibody 23-2D3 has been validated previously [24]. Biotinylated PoAb-C was used as a capture antibody, and horseradish peroxidase (HRP) -conjugated 23-2D3 Fab’ was used as a detection antibody.

Microtiter plates (96 wells) were coated by filling with 100 μL/well of 50 mM Tris-HCl buffer (pH 7.5) containing 1 μg/mL of streptavidin overnight at 4°C. Then, the plates were washed with phosphate buffered saline (PBS) and blocked with 200 μL/well of 1% (w/v) bovine serum albumin in PBS containing 0.05% NaN_3_ overnight at 4°C. After washing two times with phosphate buffered saline with tween (PBST), the wells were incubated with biotinylated PoAb-C for 1 hour at 25°C. Then, 100 μL of test samples and a known concentration of synthetic human PP peptide were added to the wells in duplicate, and the plates were incubated overnight at 4°C. After washing 4 times with PBST, 100 μL of HRP-conjugated 23-2D3 mouse IgG Fab’ was added to each well and incubated for 60 minutes at 4°C. The wells were washed with PBST 5 times, and then 100 μL of tetramethyl benzidine solution was added to each well as a substrate. Then, the plates were incubated in the dark for 30 minutes at room temperature. Reactions were terminated by adding 100 μL of 1 N H_2_SO_4_. The absorbance of the solutions at 450 nm was measured by a plate reader (E-Max; Molecular Devices, Sunnyvale, CA, USA). The concentrations of the unknown samples were calculated from the known concentrations of the synthetic human PP peptide. We also analyzed the cross-reactivities of the ELISA system with other members of the NPY family, using synthetic peptides PYY (1–42) and NPY (1–42). A recovery test was performed to assess the effects of factors in the mouse serum samples that may interfere with the reaction [29]. Synthetic human PP (1,280 pmol/L) was added to different concentrations of mouse serum samples (0%, 16.7%, and 50%), resulting in a final PP concentration of 640 pmol/L. The recovery rate was verified by the ratio of the measured concentration to the added concentration.

### Isolation of pancreatic islets

Mice were euthanized by cervical dislocation after anesthesia. After the common bile duct was clamped near the hepatic hilum, the pancreatic duct was cannulated with a 30-gauge needle, and 25 U/mL collagenase (type XI; Sigma-Aldrich, St. Louis, MO, USA) was injected. Pancreata were isolated and incubated for 30 minutes at 37°C with shaking at 85 rpm, as previously reported [30]. Islets were isolated from acinar tissue by repeated pipetting. Then, the solution was left to stand for 2 minutes so that the islets sunk to the bottom of the tube. The supernatants were collected and the rest of the solution containing the islets was moved into a dish. The islets were picked using a micropipette under a dissection microscope, and cultured in RPMI-1640 medium with 1% penicillin/streptomycin overnight.

### Measurement of PP secretion

Batches of 10 mouse islets were preincubated for 30 minutes in Krebs-Ringer bicarbonate HEPES (N-(2-hydroxyethyl)-piperazine-N’-2-ethanesulfonic acid) buffer (HKRB buffer, containing 136 mM NaCl, 10 mM HEPES [pH 7.4], 5 mM NaHCO_3_, 4.8 mM KCl, 2.5 mM CaCl_2_・2H_2_O, 1.2 mM KH_2_PO_4_, 1.2 mM MgSO_4_・7H_2_O, 2.8 mM glucose, and 0.1% bovine serum albumin). Then, batches were incubated for 60 minutes in basal (HKRB buffer) or stimulated (HKRB buffer with 16.7 mM glucose, 40 μM forskolin, 3, 10 or 30 nM GIP, 10 nM glucagon-like peptide-1 [GLP-1], and 10 μM carbachol) conditions. Concentrations of PP in the media were measured using the PP ELISA system as described above, or Mouse PPY/Pancreatic polypeptide CLIA Kit (LifeSpan BioSciences, Inc., Seattle, WA, USA) according to the manufacturer’s instructions. Insulin concentrations were measured using Mouse Insulin ELISA Kit (Morinaga Institute of Biological Science, Yokohama, Japan). Islets were transferred to 50 μL NP40 buffer (containing 150 mM NaCl, 50 mM Tris-HCl [pH 8.0], 1% NP40, and proteinase inhibitors [catalog no. 11836170001, Roche, Basel, Switzerland]) and sonicated. Then, islet protein concentrations were measured by Pierce® 660 nm Protein Assay (Thermo Fisher Scientific). PP and insulin secretion was calculated by normalizing the PP and insulin concentration of the media, respectively, by the amount of islet protein. Agonists were as follows: carbachol (Fujifilm), GIP (Peptide Institute. Inc., Osaka, Japan), GLP-1 (Peptide Institute. Inc.). Inhibitor used was nifedipine (Fujifilm).

### Immunofluorescence staining

Immunohistochemistry was performed as described previously [24]. The primary antibodies used were monoclonal mouse anti-pancreatic polypeptide (23-2D3; 0.2 μg/mL; #10501, Immuno-Biological Laboratories Co., Ltd., Gunma, Japan), guinea pig anti-insulin (Evision FLEX-Insulin, catalog no. IR002; Ready to use, Dako-Agilent, Santa Clara, CA, USA), and rabbit anti-glucagon (catalog no. ab92517; 1:2,000, Cambrige, UK). Tissue sections were incubated with a primary antibody, followed by a secondary antibody (Alexa 448 [(RRID: AB_2534117) to detect insulin; (RRID: AB_2535792) to detect glucagon, 1:2,000 each], or Alexa 594 [(RRID: AB_2534073) to detect PP, 1:2,000], Thermo Fisher Scientific) and the nuclear dye 4’,6-diamidino-2-phenylindole (DAPI; Dojindo Molecular Technologies, Kumamoto, Japan). The sections were then observed using an FV1000 confocal laser-scanning biological microscope (Olympus, Tokyo, Japan).

### Preparation of pancreatic γ cells and cytoplasmic Ca^2+^ and Na^+^ measurements

Islets were isolated from *Ppy-Clover-PEST* heterozygote mice (*Ppy*^*Clover-PEST/+*^) as described above, dispersed them into single cells and cultured overnight. Calcium flow parameters of the isolated islet cells were measured by Fura-2-acetoxymethyl (Fura 2-AM; Dojindo Molecular Technologies), and sodium flow parameters were measured by sodium-binding benzofuran isophthalate-acetoxymethyl (SBFI-AM; Thermo Fisher Scientific), as previously described [31–33]. To identify γ cells, green-fluorescent cells were specifically selected under the fluorescence microscope. To measure changes in intracellular Ca^2+^ concentrations ([Ca^2+^]_c_) in γ cells, changes in the fluorescence intensity of cells encircled in white in S1C Fig were measured. HKRB buffer was used as the solution for Ca^2+^ and Na^+^ flow. The Na^+^-free extracellular solution was prepared by removing the extracellular Na^+^, and the Na^+^ was replaced by N-Methyl-D (−) -glucamine (Fujifilm, Osaka, Japan). Agonists were as follows: carbachol, GIP, and GLP-1. Inhibitor were YM-254890 (Fujifilm) and nifedipine. The ratio of 340 nm/380 nm fluorescence was calculated, and the values were normalized to each initial value (F0), and the relative fluorescence change was referred to as F/F0. For determination of the response relationship and analyses of the effects of the inhibitors, the area under the curve was calculated.

### Statistical analyses

Statistical analysis was performed using GraphPad Prism 9.1.1 software (GraphPad Software, San Diego, CA, USA). Data are presented as the mean ± standard error of the mean (SEM). The Fisher’s test was performed to compare ratios, and the two-tailed unpaired *t*-test was used for analyzing the differences between two groups. A *p*-value of less than 0.05 was considered to indicate a statistically significant difference between groups.

## Results

### Specificity of the developed ELISA system

**Fig 1:**
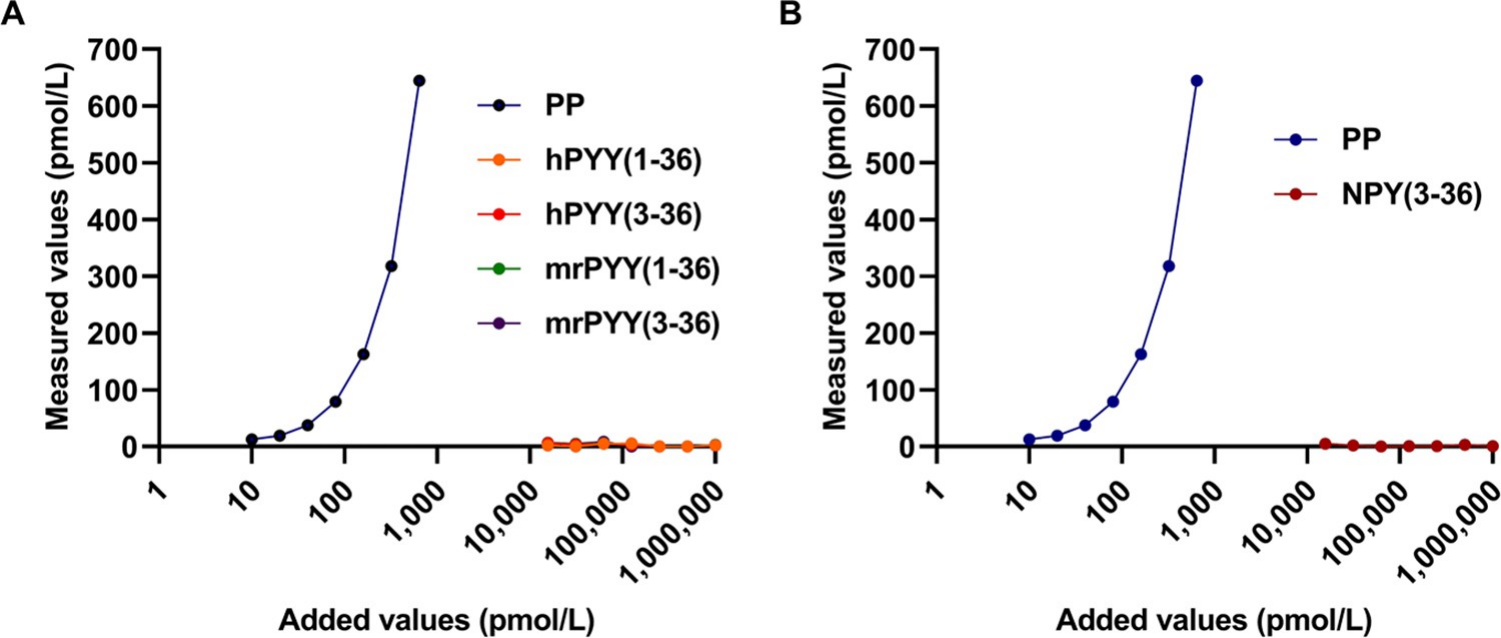
Cross-reactivities of the PP ELISA system with other NPY family peptides. PP concentrations measured by the PP ELISA system after the addition of synthetic peptides PP (A, B), PYY (A) or NPY (B) with adjusted concentrations. hPYY: Human PYY; mrPYY: Mouse and rat PYY, which have the same amino acid sequence.

To verify whether the newly established ELISA specifically detects PP, we tested the cross-reactivity of the ELISA to various synthetic peptides, including those of the NPY family (PP, PYY, and NPY). As shown in Fig 1, only the added values of PP positively correlated with the values determined by this ELISA system. In contrast to the dose-dependent activity level of PP measured using this ELISA, the activity levels of the other synthetic peptides (PYY and NPY) were negligible, even when high concentrations of samples were added. These results indicate that the PP ELISA developed in this study is highly specific to PP.

### Distribution of γ cells in pancreatic islets

The concentration of PP in mouse serum was the same level as that of the negative control using our ELISA system, suggesting that the PP concentrations may be below the detection limit of the system, and that mouse serum may contain factors that inhibit the antigen-antibody reaction. Therefore, a recovery test was performed to investigate that possibility. As a result, the recovery of 2-fold diluted serum was about 15%, and that of 6-fold diluted serum was about 48% (S1 Table). These results suggest that mouse serum may contain substances that inhibit the binding of antibodies to PP. Hence, we decided to measure the concentration of PP in the medium secreted from isolated pancreatic islets of mice.

**Fig 2:**
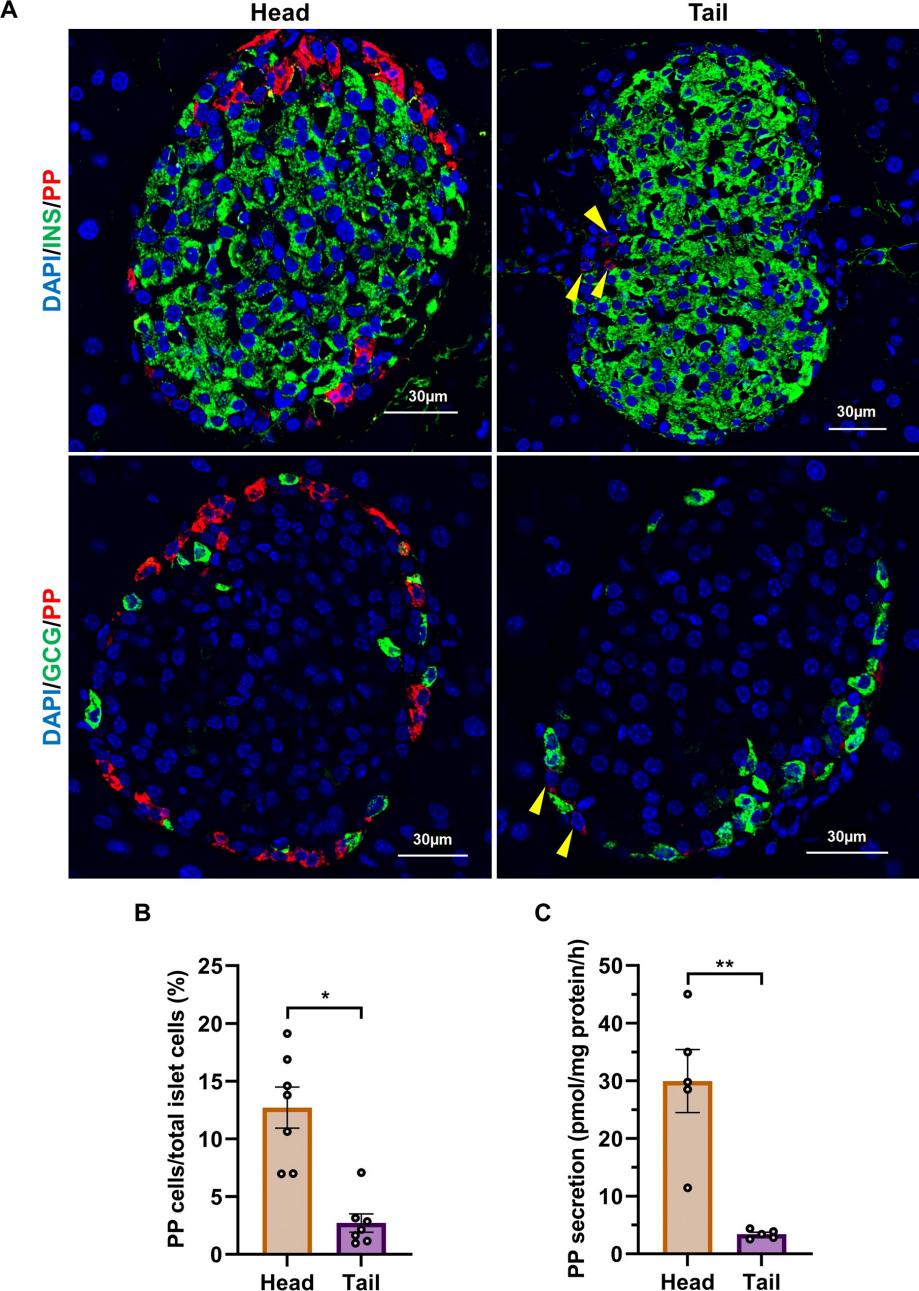
γ-cell distribution and PP secretion from islets in the head and tail of the pancreas. (A) Immunofluorescence staining of the head and tail regions of the pancreata from 10-week-old WT mice. Yellow arrowheads: γ cells in the islet of the tail region of the pancreas. Scale bars: 30 μm. (B) The ratio of γ cells in islet cells from the head and tail regions of the pancreas, isolated from 10-week-old WT mice (n = 7 mice). Data are shown as the mean ± SEM, and were analyzed by the Fisher’s test. *p < 0.05. (C) PP secretion from 10-week-old WT head and tail islets measured by the PP ELISA system (n = 5 mice). Data are shown as the mean ± SEM and analyzed by the two-tailed unpaired *t*-test. **p < 0.01.

It has been reported that γ cells are frequently found within islets of the head region of the pancreas [2,34–37], but PP antibodies used in these previous studies were not convincingly specific for PP. To determine whether γ cells are localized in the head or tail region of the pancreas, we first conducted immunostaining of the mouse pancreas using the 23-2D3 antibody [24]. γ cells were localized more frequently in the head region of the pancreas than in the tail region (Fig 2A), and quantitative analysis also showed that the number of γ cells was higher in the pancreas head than in the tail (Fig 2B), as previously reported [34–37]. We isolated islets separately from both the head and the tail of the pancreas of wild-type (WT) mice as described in S2 Fig, and the PP ELISA system was used to measure the concentration of PP secreted into the culture media from isolated islets. The amount of PP secreted from islets of the head region of the pancreas was significantly higher than that from the tail (Fig 2C). This result is consistent with the results of immunostaining (Fig 2A and 2B), and suggests that PP secretion mainly occurs from the islets located in the head of the pancreas, and that the amount of PP secretion correlates with the number of γ cells in the islets of the head or tail region of the pancreas. Therefore, we decided to use islets isolated only from the head of the pancreas in the subsequent experiments.

### Specificity and accuracy of the new PP ELISA system

**Fig 3:**
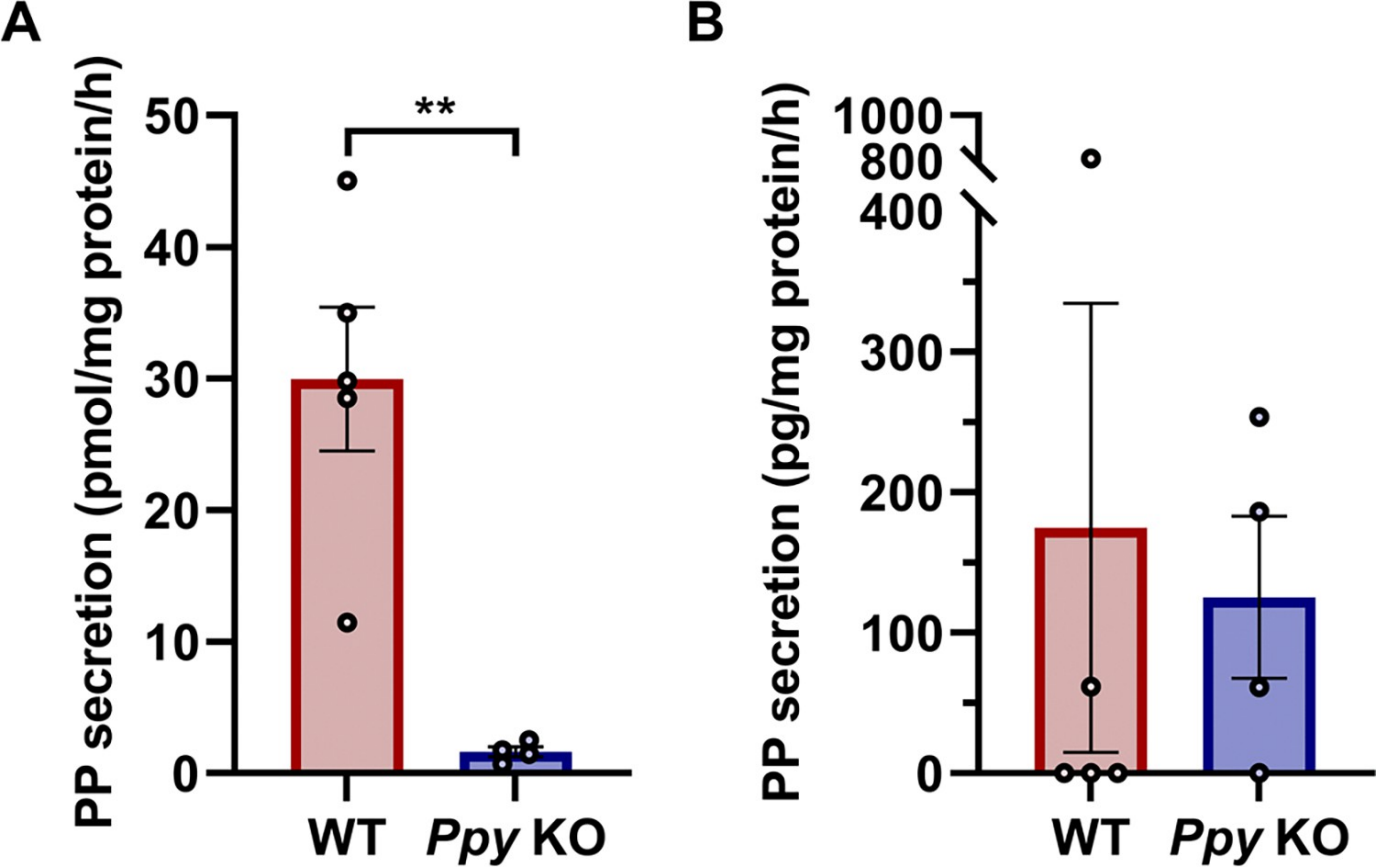
Evaluation of the accuracy of the two PP ELISA systems. (A) PP secretion from islets in the head region of the pancreas of 10-week-old WT mice (n = 5) and *Ppy*-KO mice (n = 4) measured by our PP ELISA system established in this study. (B) PP secretion from islets in the head region of the pancreas of 10-week-old WT mice (n = 5) and *Ppy*-KO mice (n = 4) measured using a commercially available kit. Values were normalized by the amount of protein in WT or *Ppy*-KO islets from the head region of the pancreas. Data are shown as the mean ± SEM, and analyzed by the two-tailed unpaired *t*-test. **p < 0.01.

Next, to investigate whether our ELISA system can accurately detect PP, islets were isolated from the head region of the pancreas of WT and *Ppy*-KO mice, and PP secretion was measured using two types of PP measurement systems; i.e., our sandwich ELISA system and a commercially available kit obtained from LifeSpan BioSciences, Inc. (catalog no. LS-F30889). PP secretion was detected from the islets of WT mice, whereas PP secretion from the islets of *Ppy*-KO mice was undetectable (Fig 3A). The commercially available kit detected PP not only in WT mouse-derived samples but also in *Ppy*-KO mouse-derived samples (Fig 3B). These results suggest that our novel ELISA system using a specific monoclonal antibody was able to accurately detect PP. On the other hand, as the commercially available system used in this study detected signals in pancreatic islet supernatants of even *Ppy*-KO mice, it is likely that signals were detected by cross-reactions with peptides other than PP.

### PP secretion is not stimulated by high concentrations of glucose

**Fig 4:**
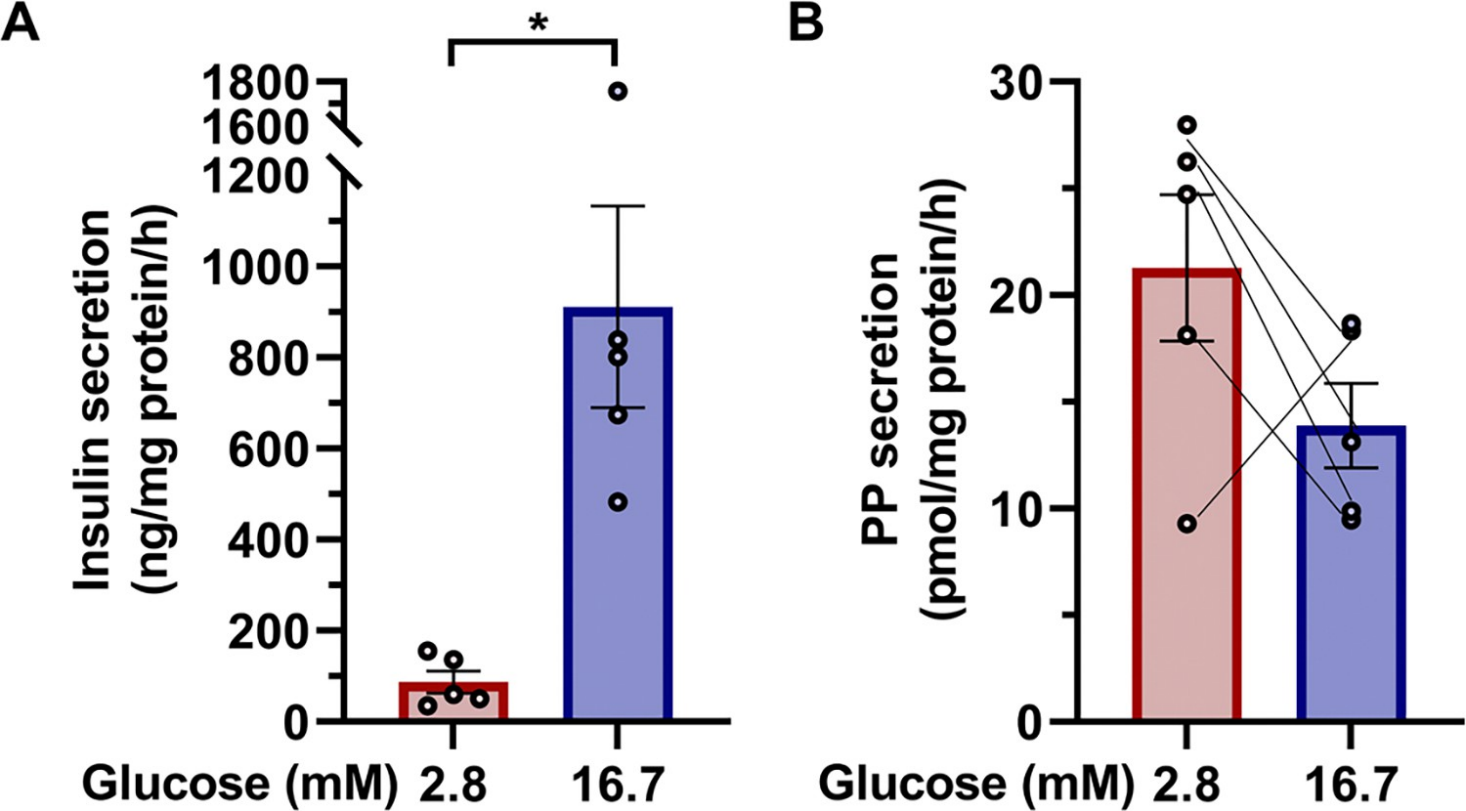
Analysis of glucose-stimulated PP secretion. Insulin (A) and PP (B) secretion from islets from the head region of the pancreas of 10-week-old WT mice, stimulated by 2.8 mM or 16.7 mM glucose for 1 hour (n = 5 mice). Values were normalized by the amount of protein from islets in the head region of the pancreas of WT mice. Data are shown as the mean ± SEM, and analyzed by the two-tailed unpaired *t*-test. *p < 0.05.

It has been reported that PP secretion is increased after diet intake [21], but the mechanism of this postprandial increase in PP secretion remains unknown, and whether PP secretion is directly stimulated by glucose or not is a matter of controversy. Therefore, our PP ELISA, which can specifically detect PP secretion, was used to evaluate PP secretion from islets exposed to a high concentration of glucose. Whereas 16.7 mM glucose induced a significant increase in insulin secretion compared with 2.8 mM glucose (Fig 4A), there was no significant difference in PP secretion in the 16.7 mM glucose group compared with that of the 2.8 mM glucose group (Fig 4B). These results suggest that PP secretion is not directly stimulated by glucose.

### PP secretion is induced by cholinergic stimulation

**Fig 5:**
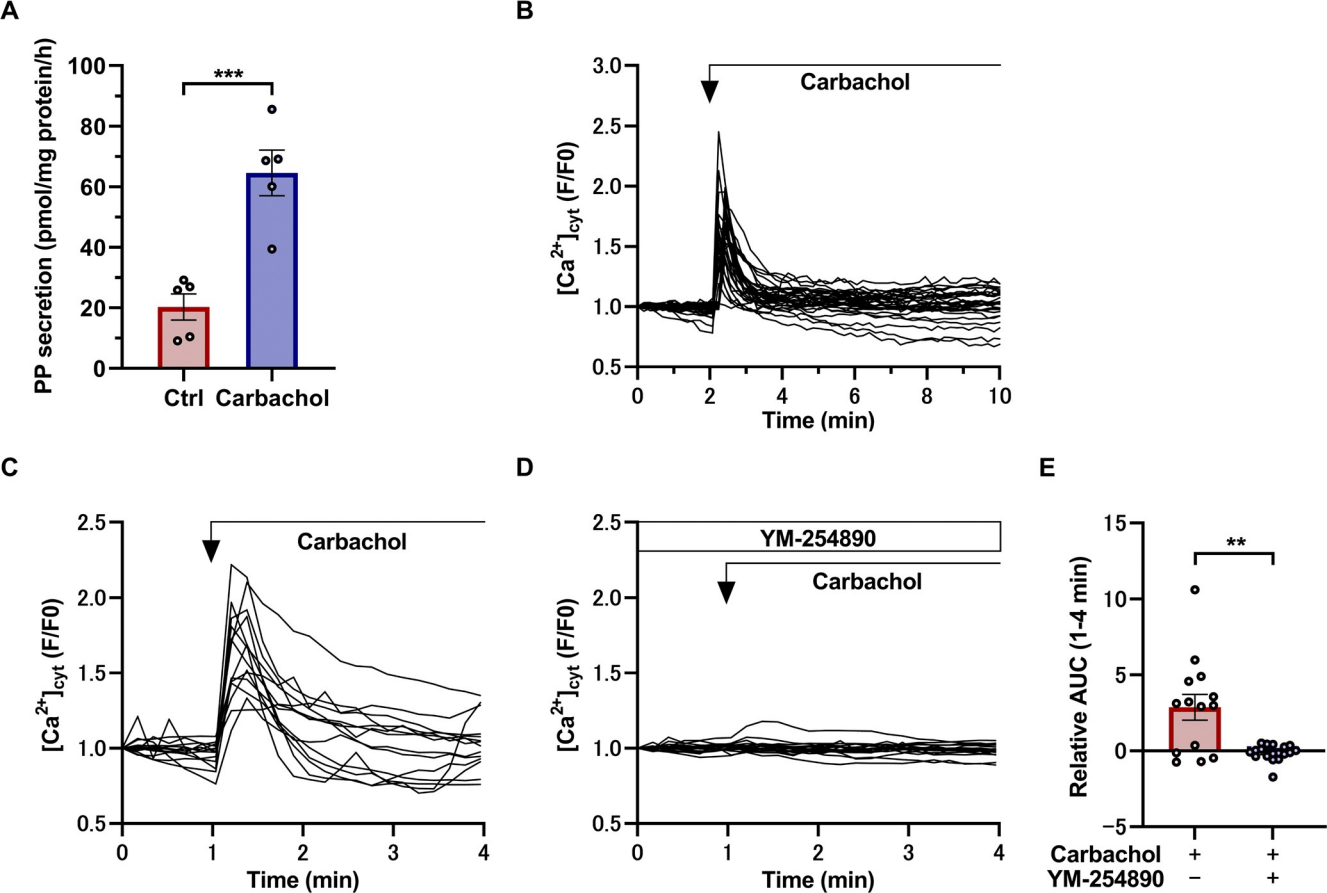
PP secretion stimulated by carbachol. (A) PP secretion from islets of the head region of the pancreas of 10-week-old WT mice, stimulated by 10 μM carbachol (n = 5 mice) for 1 hour. Values were normalized by the amount of protein from islets in the head region of the pancreas of WT mice. Data are shown as the mean ± SEM, and were analyzed by the two-tailed unpaired *t*-test. ***p < 0.001. (B) Representative time course of Ca^2+^ responses in Clover^+^ cells (n = 32 cells) from *Ppy-Clover-PEST* mice (n = 4) stimulated with 10 μM carbachol. (C, D) Representative time course of Ca^2+^ responses in Clover^+^ cells from *Ppy-Clover-PEST* mice (n = 4) stimulated with 10 μM carbachol (C, n = 14 cells), or with 10 μM carbachol and 10 μM YM-254890 (D, n = 18 cells). (E) Ca^2+^ responses to stimulation with 10 μM carbachol (n = 14 cells), or with 10 μM carbachol and 10 μM YM-254890 (n = 18 cells) in Clover^+^ cells from *Ppy-Clover-PEST* mice. Data are shown as the mean ± SEM, and were analyzed by the unpaired *t*-test. **p < 0.01. AUC: Area under the curve.

Similar to insulin secretion from β cells, as PP secretion may be induced by postprandial vagal stimulation [38], we investigated whether PP secretion is increased by carbachol, a cholinergic agonist, using our ELISA system. Our results showed that PP secretion from mouse islets stimulated with 10 μM carbachol was significantly increased compared with the control (Fig 5A). To investigate the intracellular signaling of PP secretion induced by carbachol, we studied its effects on [Ca^2+^]_c_ in γ cells obtained from *Ppy-Clover-PEST* mice, in which *Clover-PEST* is expressed under the control of the endogenous *Ppy* promoter (S1A Fig). The γ cells of *Ppy-Clover-PEST* mice demonstrated green fluorescence when exposed to 488 nm excitation light, making it possible to identify the γ cells by microscopy (S1B Fig). In Clover^+^ cells, 10 μM carbachol induced a transient increase in [Ca^2+^]_c_ (Fig 5B). This increase in Ca^2+^ by carbachol was inhibited by the selective Gαq/11 protein inhibitor YM-254890 (Fig 5C–5E), suggesting that there may be a Gαq/11 signaling pathway that activates PP secretion in γ cells as well as in β cells [39,40].

### PP secretion is stimulated by GIP, but not by GLP-1

**Fig 6:**
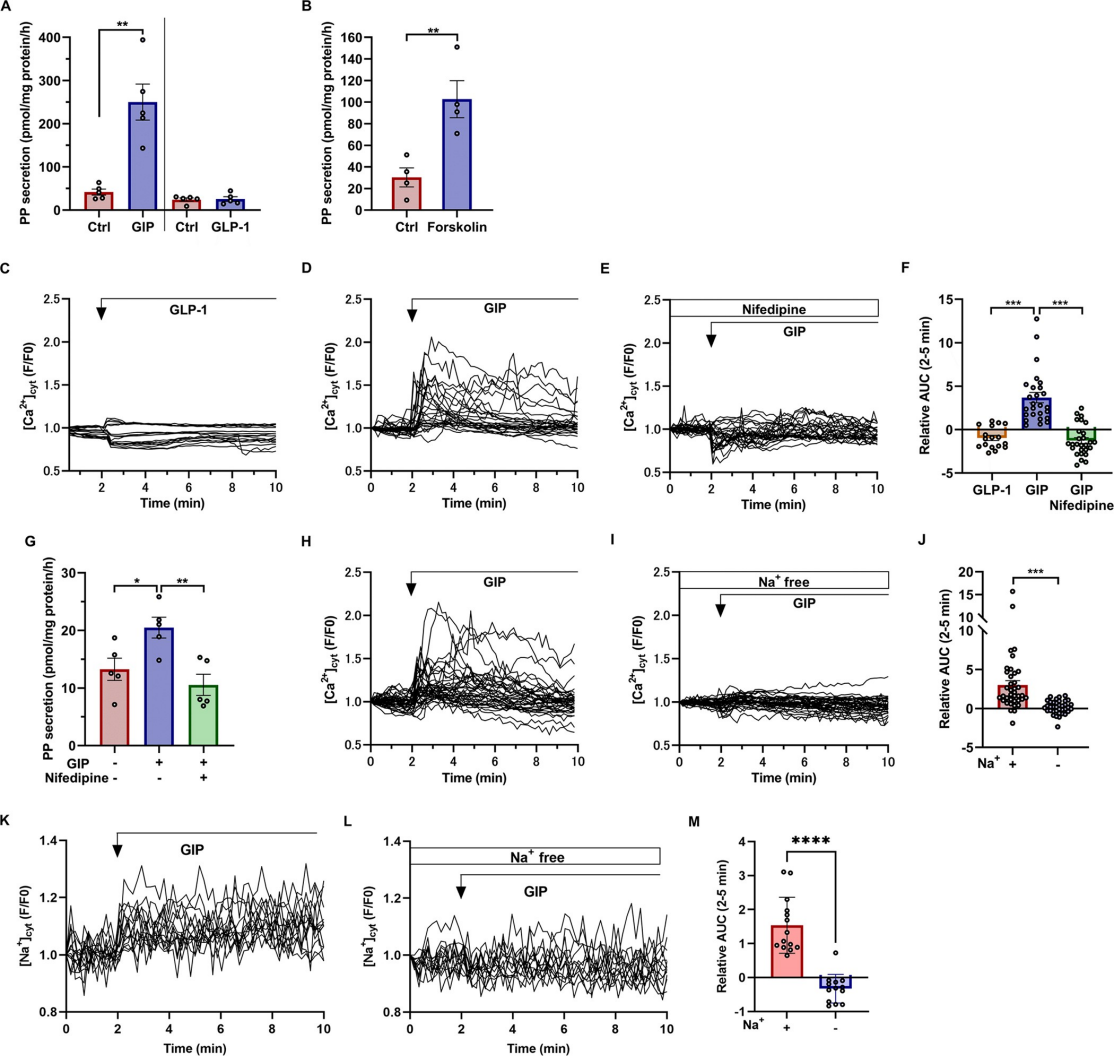
GIP-stimulated PP secretion. (A) PP secretion from islets in the head region of the pancreas of 10-week-old WT mice stimulated with 10 nM GIP (n = 5 mice, left side), or 10 nM GLP-1 (n = 5 mice, right side) for 1 hour. (B) PP secretion from islets in the head region of the pancreas of 8 to 13-week-old WT mice stimulated with 40 μM forskolin (n = 4 mice). (C-E) Representative time courses of Ca^2+^ responses in Clover^+^ cells from *Ppy-Clover-PEST* mice (n = 4) treated with 10 nM GLP-1 (C, n = 17 cells), 10 nM GIP (D, n = 26 cells), or 10 nM GIP and 10 μM nifedipine (E, n = 27 cells). (F) Ca^2+^ responses to stimulation with 10 nM GLP-1 (n = 17 cells), 10 nM GIP (n = 26 cells), or 10 nM GIP and 10 μM nifedipine (n = 27 cells) in Clover^+^ cells from *Ppy-Clover-PEST* mice. (G) PP secretion from islets of the head region of the pancreas of 8 to 13-week-old WT mice, stimulated with 10 nM GIP, with or without 10 μM nifedipine (n = 5). (H, I) Representative time course of Ca^2+^ responses in Clover^+^ cells from *Ppy-Clover-PEST* mice (n = 4) stimulated with 10 nM GIP in HKRB buffer with (H, n = 38 cells) or without (I, n = 37 cells) Na^+^. (J) Ca^2+^ responses to 10 nM GIP stimulation in HKRB medium with or without Na^+^, in Clover^+^ cells from *Ppy-Clover-PEST* mice. (K, L) Representative time courses of Na^+^ responses in Clover^+^ cells from *Ppy-Clover-PEST* mice (n = 4) treated with 10 nM GIP in HKRB buffer with (K, n = 14 cells) or without (L, n = 13 cells) Na^+^. (M) Na^+^ responses to 10 nM GIP stimulation in HKRB medium with or without Na^+^, in Clover^+^ cells from *Ppy-Clover-PEST* mice. PP secretion values were normalized by the amount of protein in islets from the head region of the pancreas. Data are shown as the mean ± SEM, and were analyzed by the unpaired *t*-test. *p < 0.05, **p < 0.01, ***p < 0.001; AUC: Area under the curve.

GIP and GLP-1 are secreted from K and L cells in the small intestine, respectively, and their secretion is enhanced by oral glucose administration, but not by intravenous glucose infusion [41]. Therefore, incretin is expected to stimulate PP secretion postprandially. Although it has been reported that GIP induces PP secretion in humans, mice, and pigs [19,20], it is not clear whether GIP acts directly on γ cells to stimulate PP secretion. Next, we analyzed whether GIP and GLP-1 stimulate PP secretion. Compared with the control group, PP secretion was markedly increased in the group stimulated with 10 nM GIP (Fig 6A, left panel). GIP-stimulated PP secretion was observed in a dose-dependent manner (S3 Fig). In contrast, as shown in the right panel of Fig 6A, PP secretion in the group stimulated with 10 nM GLP-1 was similar to that in the control group. These data suggest that GIP stimulates PP secretion, whereas GLP-1, which shows a postprandial increase in the blood circulation similarly to GIP, does not induce PP secretion.

It is well known that GIP acts in many tissues through cyclic adenosine monophosphate (cAMP) activation [42]. Therefore, we next analyzed whether forskolin, a reagent that enhances cAMP production, induces PP secretion. Compared with the control group, PP secretion was significantly enhanced in the group exposed to 40 μM forskolin (Fig 6B). These data suggest that enhanced cAMP production may be involved in the induction of PP secretion and GIP, may be an endogenous agonist that elicits PP secretion.

To investigate the intracellular signaling that elicits PP secretion, we studied the effects of GIP and GLP-1 on [Ca^2+^]_c_ in γ cells obtained from *Ppy-Clover-PEST* mice. In Clover^+^ cells, the increase in [Ca^2+^]_c_ was not induced by GLP-1, which did not induce PP secretion (Fig 6C and 6F). In contrast, 10 nM GIP induced an increase in [Ca^2+^]_c_ (Fig 6D and 6F); the GIP-induced increase in [Ca^2+^]_c_ was completely suppressed by 10 μM nifedipine (Fig 6E and 6F). Consistent with the change in [Ca^2+^]_c_ in γ cells, PP secretion from mouse islets stimulated by 10 nM GIP was suppressed by 10 μM nifedipine (Fig 6G). These results suggest that GIP, which induces PP secretion, increases [Ca^2+^]_c_ in γ cells through the activity of L-type Ca^2+^ channels.

Depolarization of the cell membrane is essential for the activation of voltage-gated Ca^2+^ channels (VDCCs), but it was unclear as to how GIP depolarizes the cell membrane. To clarify whether Na^+^ entry is involved, we analyzed whether there was a difference in the change in [Ca^2+^]_c_ when Clover^+^ cells were stimulated with 10 nM GIP, depending on the presence or absence of Na^+^ in the HKRB buffer used for the measurement. [Ca^2+^]_c_ of Clover^+^ cells was increased by 10 nM GIP (Fig 6H and 6J). In contrast, in Na^+^-depleted HKRB buffer, [Ca^2+^]_c_ of Clover^+^ cells was not altered by 10 nM GIP (Fig 6I and 6J). To investigate the Na^+^ influx into γ cells by GIP stimulation, intracellular Na^+^ concentrations ([Na^+^]_c_) in Clover^+^ cells isolated from *Ppy-Clover-PEST* mice were measured. [Na^+^]_c_ of Clover^+^ cells were increased by 10 nM GIP (Fig 6K and 6M). In contrast, in Na^+^-depleted HKRB buffer, [Na^+^]_c_ of Clover^+^ cells were not altered by 10 nM GIP (Fig 6L and 6M). These results indicate that GIP induces Na^+^ entry into γ cells, which in turn causes depolarization of the cell membrane.

Finally, we investigated the reason as to why GLP-1 and GIP demonstrate different PP secretion activities. One possibility is the different expression levels of the Glp1 receptor *(Glp1r)* and Gip receptor *(Gipr)* in γ cells, and hence we analyzed the datasets of single-cell RNA sequence analysis, which have already been deposited by the Tabula Muris consortium [43]. C57BL/6 mouse islet cells were classified into nine clusters according to their cellular identity, and pancreatic β cells expressed both *Glp1r* and *Gipr* at substantial levels, although γ cells expressed much less *Glp1r* than *Gipr* (S4 Fig). These results may hence explain the lack of PP secretion in response to GLP-1.

## Discussion

In this study, we developed a new PP sandwich ELISA using our recently developed monoclonal antibody that is specific for PP [24]. This sandwich ELISA detects PP from islet supernatants of WT mice, but not from islet supernatants of *Ppy*-KO mice, suggesting that our ELISA system is highly specific for PP. Antibody-based assays, such as RIA, competitive ELISA, and sandwich ELISA have been used to measure PP secretion from isolated mouse islets [16,23]. However, in most of these PP detection systems, the specificity of the PP antibodies had not been validated, and hence there remained a possibility that peptides other than PP were being detected. In support of this possibility, the commercially available sandwich ELISA used in the present study detected a “pseudo” PP signal in supernatants obtained from *Ppy*-KO islets, which was equivalent to the signal detected in supernatants from WT islets.

Glucose has been reported to stimulate PP secretion from isolated islets [23], but conversely, it has also been reported that PP secretion is not induced by glucose [16]. We hence investigated whether PP secretion is stimulated by glucose in isolated mouse islets, using our PP ELISA. Our results showed that PP secretion was not induced by 16.7 mM glucose, suggesting that the secretion of PP is not directly regulated by glucose, and it may not be regulated by insulin or other hormones that are secreted from the islets in response to glucose.

Currently, the lack of γ cell lines makes it difficult to elucidate the mechanism of intracellular signaling by agonists involved in PP secretion. There have been several reports on the regulation of Ca^2+^ in γ cells using isolated pancreatic islets [22,44], but all of these studies used indirect methods to detect PP-expressing cells. In the present study, we established a system to directly monitor PP-expressing cells by developing mice in which exon 2, the coding region of the PP protein in the *Ppy* locus, was replaced by the fluorescent protein Clover. Because the excitation wavelength of Fura-2 (340 nm, 380 nm) is different from that of the Clover protein (505 nm), it is possible to observe the fluorescences of both Fura-2 and the Clover protein simultaneously. Using this system, we observed a transient and dynamic intracellular Ca^2+^ increase induced by carbachol, which stimulates PP secretion (Fig 5B). In addition, the carbachol-induced increase in Ca^2+^ was inhibited by YM-254890 (Fig 5C–5E). These data indicated that the Ca^2+^ signaling mechanism elicited by carbachol-induced PP secretion in γ cells is mediated by Gαq/11.

In the present study, we analyzed GIP and GLP-1 as candidate endogenous ligands for PP secretion. Our results showed that PP secretion was induced only by GIP, but not by GLP-1 (Fig 6A). Consistent with this result, the increase in Ca^2+^ was induced only by GIP, but not by GLP-1 (Fig 6C and 6D). Furthermore, this Ca^2+^ increase was abrogated by nifedipine, an inhibitor of VDCCs (Fig 6E). Importantly, GIP-induced PP secretion was also inhibited by nifedipine (Fig 6G). In β cells, GIP activates the amplifying pathway that promotes insulin secretion [45], and a high glucose concentration is required for GIP-mediated insulin secretion. In addition, it has been reported that GIP induces glucagon secretion in α cells in the presence of high concentrations of amino acids [46]. In contrast, in γ cells, GIP stimulates PP secretion in HKRB containing 2.8 mM glucose (Fig 6A). Pharmacological studies using Na^+^-depleted culture medium suggested that in γ cells, in the presence of HKRB containing 2.8 mM glucose, GIP stimulates Na^+^ influx into the cells and depolarizes the cell membrane, thereby activating VDCCs and inducing Ca^2+^ influx into the cells (Fig 6). This suggests the existence of a GIP-mediated Ca^2+^ signaling mechanism that is characteristic of γ cells, but the molecular details of this mechanism remain unclear. Single-cell RNA-sequence data of mice pancreatic islets showed that the expression of *Glp1r* is very low in γ cells (S4 Fig), presumably accounting for the unresponsiveness of g cells to GLP-1.

The limitation of this study is that the ELISA system that we developed is not able to measure PP concentrations in the plasma and sera of mice. The results of a recovery test suggested that the serum may contain unknown substances that inhibit the binding of PP to its antibodies (S1 Table). If we can establish a system to measure PP concentrations in serum by excluding proteins that inhibit PP antibody binding, for example, by using columns, this will increase our understanding of the physiological functions of PP. In addition, it has been suggested that PP is degraded by dipeptidyl peptidase IV and neprilysin [47], and hence the use of inhibitors of PP-degrading enzymes during serum collection is expected to stabilize PP.

In conclusion, we developed an ELISA that specifically recognizes PP using a highly specific PP antibody [24]. We also succeeded in specific monitoring PP-expressing cells in real time using Clover^+^ cells prepared from *Ppy-Clover-PEST* mice. Using these systems, we identified agonists, such as carbachol and GIP, which stimulate PP secretion, and clarified the involvement of Ca^2+^ signaling in their secretory pathways.

## Supporting information

S1 Fig Generation of *Ppy-Clover-PEST* mice. (A) *Ppy-Clover-PEST* knock-in vector was constructed by inserting a *Clover-PEST* sequence (PEST sequence derived from mouse ornithine decarboxylase was fused to the C-terminal region of Clover) into the same *Ppy* gene locus and generated mice. (B) Immunofluorescence staining of the head regions of the pancreata from 10-week-old *Ppy-Clover-PEST* mice. Scale bars: 30 μm. (C) Isolated islet cells excited by 340 nm light (left panel) and *Clover*-expressing cells excited by 488 nm light (right panel). The two panels show the same field of view. Scale bars: 50 μm.
(TIF)

S2 Fig Schematic diagram of the head and tail of a mouse pancreas. The pancreas was divided into two parts, i.e., the head and tail, at the dotted line as depicted.
(TIF)

S3 Fig Dose-dependency of GIP-stimulated PP secretion. PP secretion from islets of the head region of the pancreas of 10-week-old WT mice, stimulated with 3, 10, or 30 nM GIP (n = 3 mice each) for 1 hour. Values were normalized by the amount of protein in the islets of the head region of the pancreas of WT mice. Data are shown as the mean ± SEM.
(TIF)

S4 Fig Gene expression in γ cells. (A) tSNE visualization of pancreatic cells analyzed using single-cell RNA sequence analysis datasets [41]. Feature plots and violin plots of *Glp1r* (B) and *Gipr* (C) mRNA expression in various pancreatic cell clusters.
(TIF)

S1 Table Recovery rates of human PP from mouse serum. Equal amounts of synthetic human PP (640 pmol/L) were dissolved in solutions with different serum contents (50%, 16.7%, and 0%). The recovery rate (%) of PP from each solution (n = 3 mice) was calculated from the measured values (the mean value of duplicate samples) and the added values.
(TIF)
