# Supplementary figures and images for: Establishment of an enzyme-linked immunosorbent assay for mouse pancreatic polypeptide clarifies the regulatory mechanism of its secretion from pancreatic γ cells
Source: PLoS One. 2022 Aug 17;17(8):e0269958. doi: 10.1371/journal.pone.0269958 (PMC9385059; doi:10.1371/journal.pone.0269958)

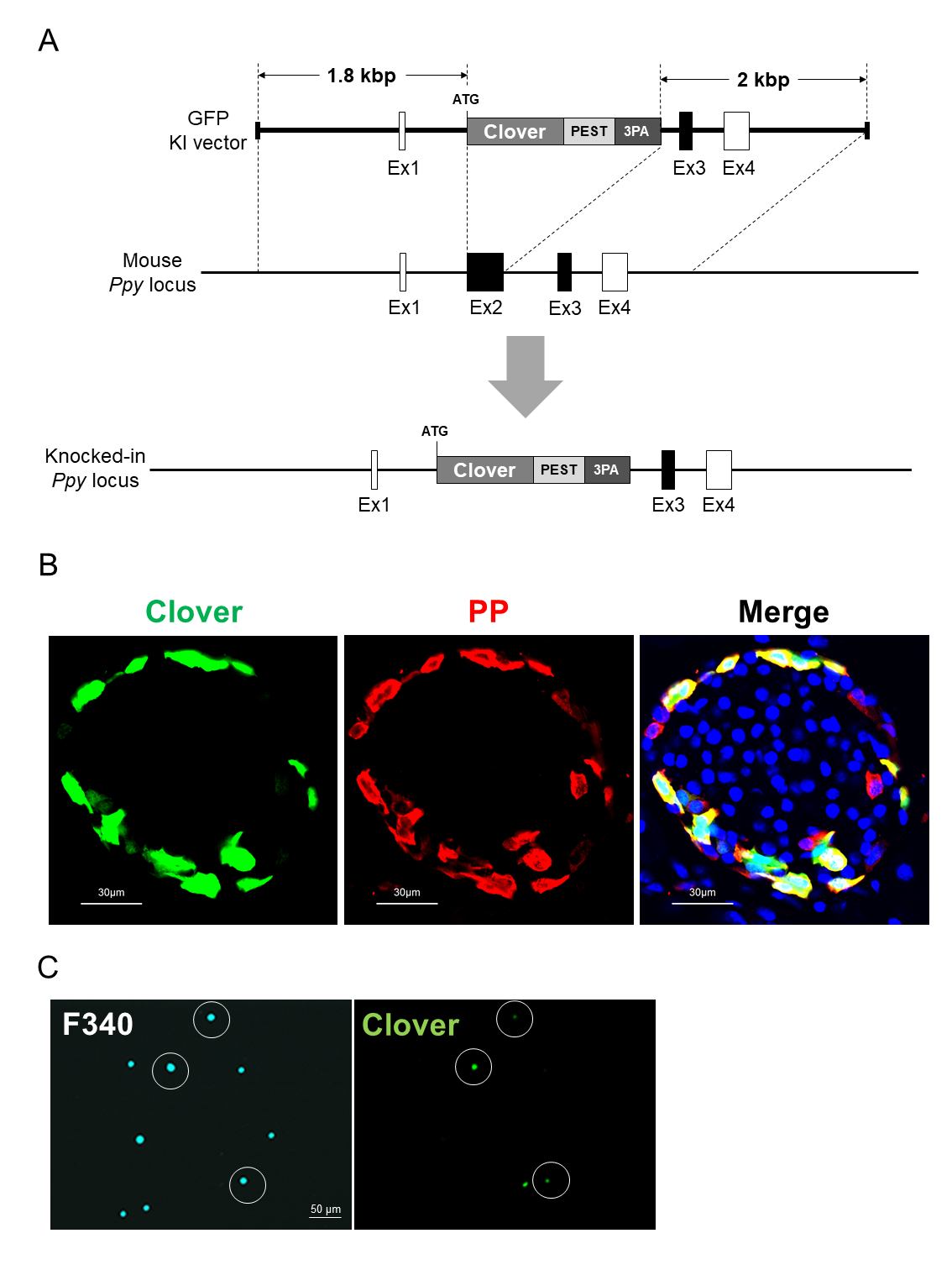

Supplement: S1 Fig — (A) Ppy-Clover-PEST knock-in vector was constructed by inserting a Clover-PEST sequence (PEST sequence derived from mouse ornithine decarboxylase was fused to the C-terminal region of Clover) into the same Ppy gene locus and generated mice. (B) Immunofluorescence staining of the head regions of the pancreata from 10-week-old Ppy-Clover-PEST mice. Scale bars: 30 μm. (C) Isolated islet cells excited by 340 nm light (left panel) and Clover-expressing cells excited by 488 nm light (right panel). The two panels show the same field of view. Scale bars: 50 μm. (TIF) [file pone.0269958.s001.tif]

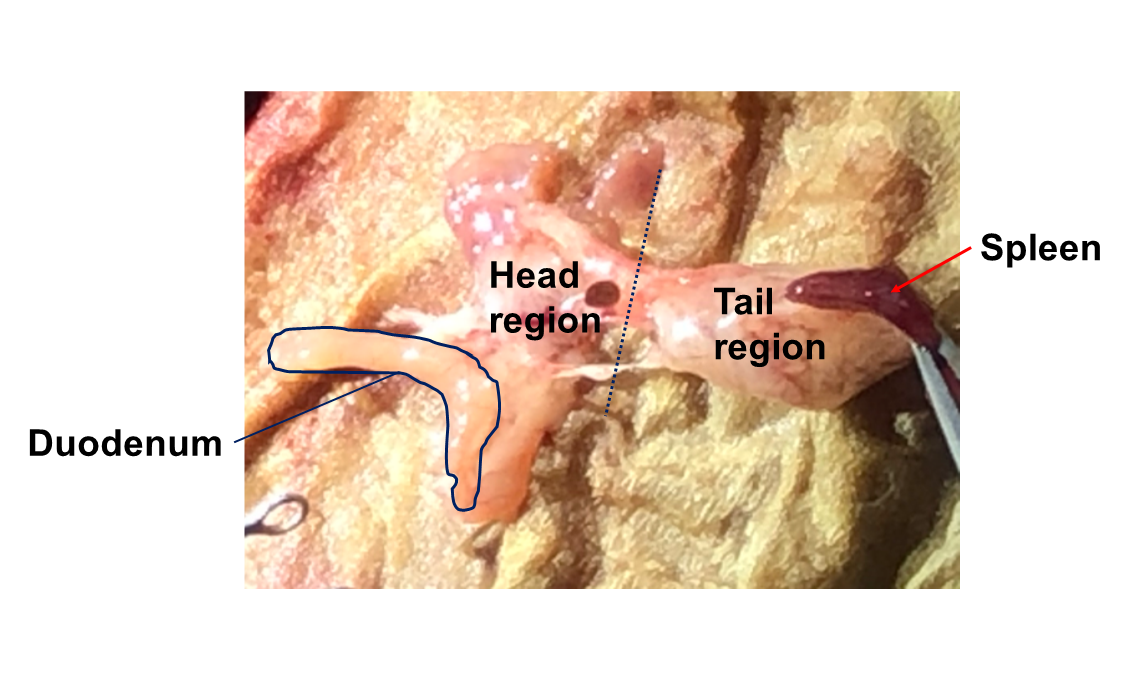

Supplement: S2 Fig — The pancreas was divided into two parts, i.e., the head and tail, at the dotted line as depicted. (TIF) [file pone.0269958.s002.tif]

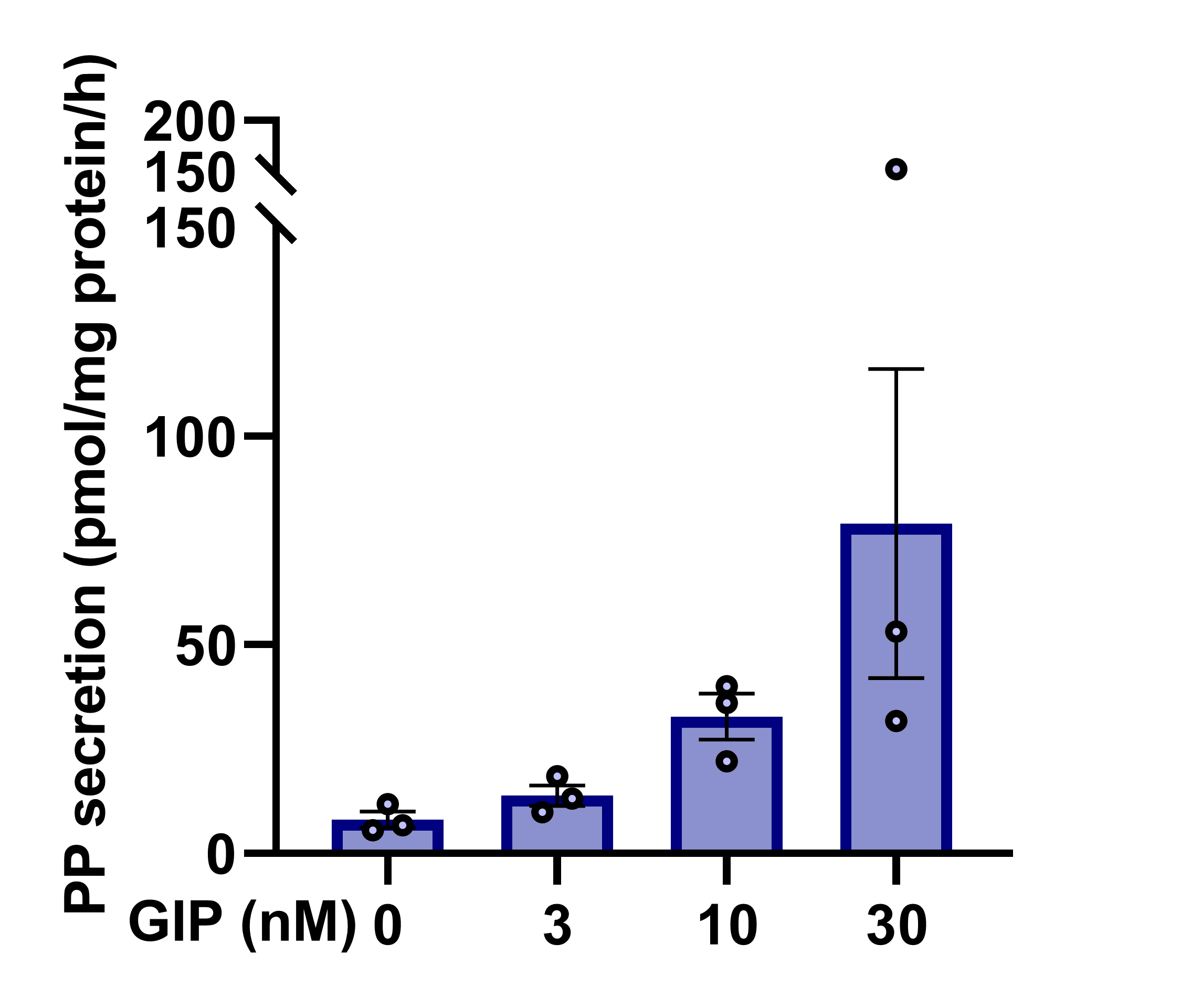

Supplement: S3 Fig — PP secretion from islets of the head region of the pancreas of 10-week-old WT mice, stimulated with 3, 10, or 30 nM GIP (n = 3 mice each) for 1 hour. Values were normalized by the amount of protein in the islets of the head region of the pancreas of WT mice. Data are shown as the mean ± SEM. (TIF) [file pone.0269958.s003.tif]

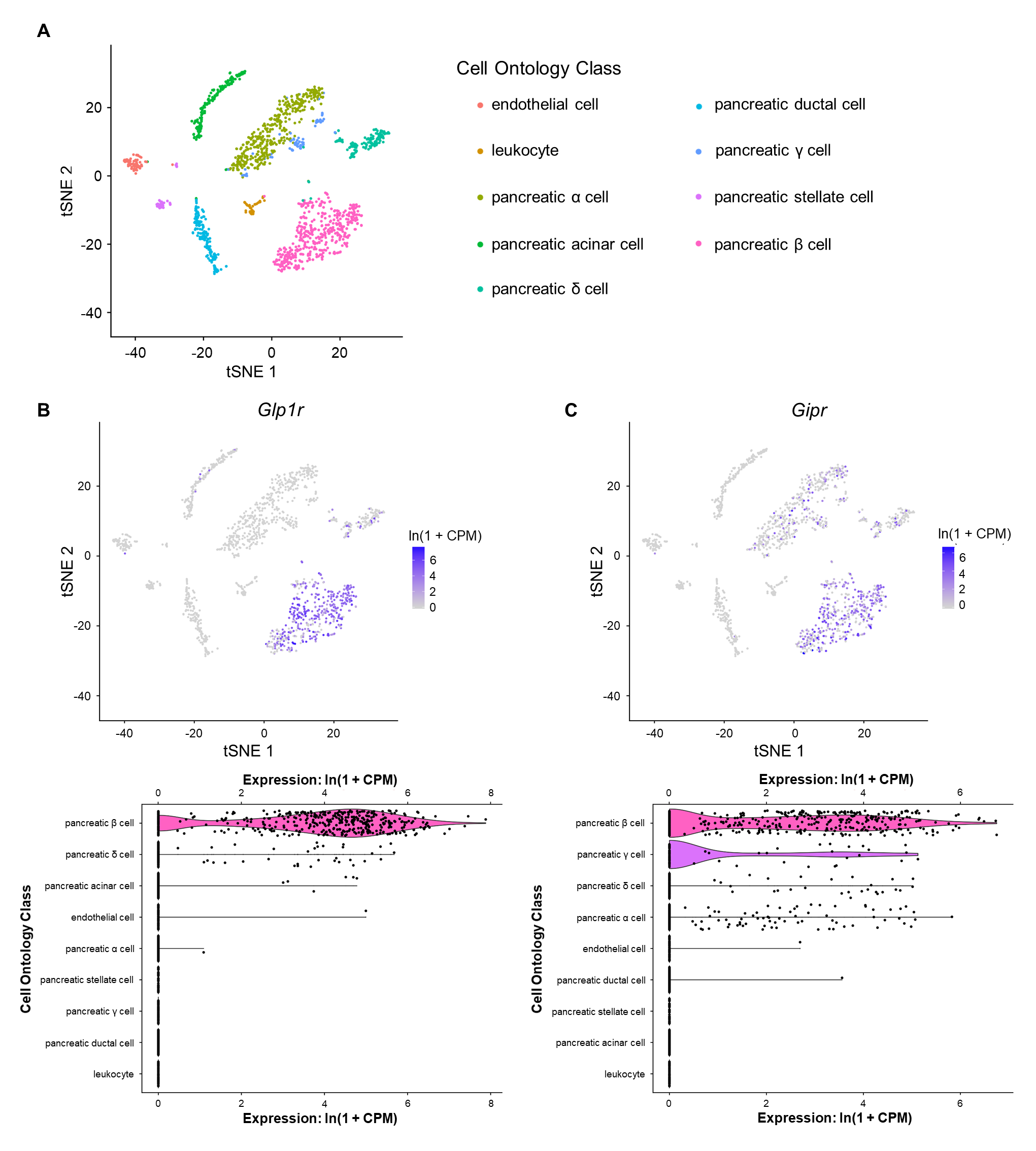

Supplement: S4 Fig — (A) tSNE visualization of pancreatic cells analyzed using single-cell RNA sequence analysis datasets [41]. Feature plots and violin plots of Glp1r (B) and Gipr (C) mRNA expression in various pancreatic cell clusters. (TIF) [file pone.0269958.s004.tif]

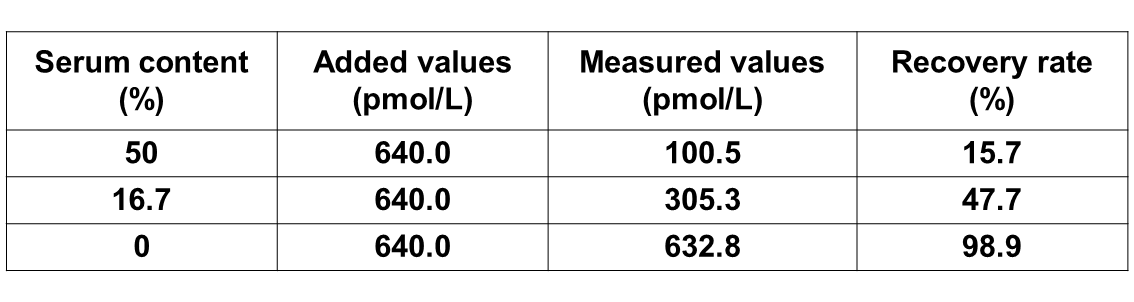

Supplement: S1 Table — Equal amounts of synthetic human PP (640 pmol/L) were dissolved in solutions with different serum contents (50%, 16.7%, and 0%). The recovery rate (%) of PP from each solution (n = 3 mice) was calculated from the measured values (the mean value of duplicate samples) and the added values. (TIF) [file pone.0269958.s005.tif]
